# A Quasi-Experimental Assessment of the Effect of the 2009 WIC Food Package Revisions on Breastfeeding Outcomes

**DOI:** 10.3390/nu15020414

**Published:** 2023-01-13

**Authors:** Kelly Kogan, Priyanka Anand, Sina Gallo, Alison Evans Cuellar

**Affiliations:** 1 Department of Health Administration and Policy, College of Public Health, George Mason University, Fairfax, VA 22030, USA; 2 Department of Nutritional Sciences, College of Family and Consumer Sciences, University of Georgia, Athens, GA 30602, USA

**Keywords:** Special Supplemental Nutrition Program for Women, Infants, and Children (WIC), breastfeeding, difference-in-differences (DID), propensity scores, intent-to-treat analysis, nutrition policy, federal food assistance policy, causal inference

## Abstract

Breastfeeding rates among infants participating in the Special Supplemental Nutrition Program for Women, Infants, and Children (WIC) are consistently lower than those of WIC nonparticipants. The 2009 WIC food package revisions were intended to incentivize breastfeeding among the WIC population. To examine the effectiveness of this policy change, we estimated an intent-to-treat regression-adjusted difference-in-difference model with propensity score weighting, an approach that allowed us to control for both secular trends in breastfeeding and selection bias. We used novel data from the Feeding Infants and Toddlers Survey from 2008 and 2016. We defined our treatment group as infants eligible for WIC based on household income and our control group as infants in households with incomes just above the WIC eligibility threshold. The breastfeeding outcomes we analyzed were whether the infants were ever breastfed, breastfed through 6 months, and breastfed exclusively through 6 months. We observed significant increases in infants that were ever breastfed in both the treatment group (10 percentage points; *p* < 0.01) and the control group (15 percentage points; *p* < 0.05); however, we did not find evidence that the difference between the two groups was statistically significant, suggesting that the 2009 revisions may not have had an effect on any of these breastfeeding outcomes.

## 1. Introduction

The Special Supplemental Nutrition Program for Women, Infants, and Children (WIC) is a federally funded, state-administered program that provides supplemental foods, health care referrals, nutrition education, and breastfeeding support to low-income women, infants, and children up to age five. Studies have shown that women and children who participate in WIC have better health outcomes compared to low-income women and children who do not participate in WIC. For example, prenatal WIC participation is associated with fewer premature births, fewer infants born with low birth weight or small for gestational age, and fewer infant deaths [1,2,3,4]. WIC participation may also contribute to improvements in child immunization status [5], maternal and child diet quality [5], and access to healthy food [6,7,8]. Additionally, WIC is cost-effective; a recent study using simulations of WIC participants in California estimated savings of nearly $2.50 in medical, educational, and productivity costs for every $1 invested in prenatal WIC services [9].

WIC’s impact on another key metric of public health—the initiation, duration, and exclusivity of breastfeeding—is less clear. It is widely recognized that breastfeeding confers numerous health benefits on mothers and infants alike [10,11,12,13]. However, WIC participants have consistently lagged behind both eligible and ineligible nonparticipants in numerous breastfeeding behaviors [14,15,16]. This disparity has led to concerns that WIC’s provision of free infant formula as part of its supplemental food benefit (referred to as a “food package”) may encourage enrollment by women who have already decided to formula-feed [17,18]. Alternatively, WIC participation may disincentivize breastfeeding due to the significantly higher market values of the food packages for formula-fed infants compared to the market value of the food package for fully breastfeeding women [17,19]. Partially in response to the latter concern, in 2009 the U.S. Department of Agriculture (USDA) revised WIC’s infant and maternal food packages [20] based on a 2005 proposal [19] from the Institute of Medicine (IOM; later renamed the Academy of Medicine), intending to reduce these differences through adjustments to the types and quantities of the included foods. However, due to the requirement that the aggregate costs of the revised food packages not exceed those of the food packages they were intended to replace [20], the 2009 revisions did not fully eliminate the gap in market values that were a focus of these revisions [19].

There is substantial policy interest in assessing whether the 2009 WIC food package revisions achieved the intended effect of increasing the rates of breastfeeding behaviors among WIC participants. Three studies conducted shortly after the implementation of the 2009 revisions suggested that the effect was positive. Two were case studies involving data from WIC agencies [21] and WIC participants [22] in southern California. The third relied on administrative data from a national random sample of 17 local WIC agencies in 10 states [23]. For the purposes of policy assessment, a major limitation of these studies was their use of a pre–post design that lacked a non-WIC comparison group, making it difficult to distinguish overall trends in breastfeeding behaviors among low-income women from changes caused by the 2009 food package revisions. The lack of a comparison group also prevented the researchers from addressing the potential for biased estimates due to selection bias. This form of bias occurs when participants in a program (such as WIC) systematically differ from nonparticipants in ways that are associated with an outcome (such as breastfeeding). Numerous studies assessing the effectiveness of WIC have documented the presence of selection bias from negatively selected observable [3,24] and likely unobservable [25,26] characteristics.

Several more recent studies examining the effect of the 2009 WIC food package provisions on breastfeeding outcomes have used models that included a comparison group, with mixed results. Joyce and Reeder [27] fit several models comparing changes in breastfeeding behaviors among WIC participants and low-income nonparticipants using a comparative interrupted time series design. Bersak and Sonchak-Ardan [28] relied upon a difference-in-differences (DID) model to examine pre–post differences in breastfeeding initiation among WIC-participating mothers relative to differences among Medicaid-eligible WIC-nonparticipating mothers. In both instances, the researchers concluded that the revised WIC food packages did not have the desired effect due to the presence of improvements in breastfeeding outcomes among all mothers, WIC and non-WIC alike. The only study that found a positive effect from the revisions was by Li et al. [29]. Here, the researchers compared breastfeeding outcomes among WIC participants and income-eligible nonparticipants using propensity score matching, a technique that is used to reduce selection bias by conditioning on observed differences between comparison groups [30]. However, the researchers were unable to account for selection bias caused by unobserved differences between the two groups.

The aim of the current study was to contribute to the prior literature by examining whether the 2009 WIC food package revisions led to increases in several breastfeeding behaviors using novel data and a study design that addressed the methodological limitations of prior work. Our data were from the Feeding Infants and Toddlers Survey [31,32], which to our knowledge has not been used in any previous analysis of the effect of the 2009 WIC food package revisions on breastfeeding outcomes. Our design consisted of an intent-to-treat (ITT) analysis with a regression-adjusted DID estimator and propensity score weighting [33]. This combination of methods allowed us to distinguish between treatment effects and overall trends in breastfeeding while more effectively addressing the potential for selection bias.

## 2. Materials and Methods

### 2.1. Study Design

To address the study aim, we conducted a secondary data analysis using a quasi-experimental approach consisting of a DID model, an ITT analysis, and propensity score weighting for each of our breastfeeding outcomes. DID models are frequently used to estimate the effect of a new policy by comparing pre–post changes in outcomes in a group affected by the newly implemented policy (the treatment group) to pre–post changes in outcomes in a group unaffected by the policy (the control group) [34]. In this study, we used a DID model to estimate the impact of the 2009 WIC food package revisions on breastfeeding outcomes by comparing average changes in breastfeeding behaviors at two time points (one before the revisions in 2008 and the other after the revisions in 2016) among each of the groups being compared.

We implemented the ITT analysis according to how we defined our treatment group. Specifically, we chose infants that were eligible for WIC based on household income (i.e., ≤185% of the federal poverty level (FPL)) as our treatment group rather than infants who were WIC participants [30]. This approach allowed us to address the potential for selection bias due to systematic differences between infants whose mothers chose to enroll them in WIC and infants whose mothers chose not to enroll them in WIC. For the control group, we chose infants whose household incomes were just above the WIC eligibility threshold since they were likely to be similar to the treatment group in both observable and unobservable characteristics, except for the fact that they were precluded from enrolling in WIC. In addition, infants in the control group were likely to experience the same secular trends related to breastfeeding behaviors as infants in the treatment group. Finally, we incorporated propensity score weights into the analysis to further reduce selection bias [33].

### 2.2. Data Source

The Feeding Infants and Toddlers Study (FITS) is a series of cross-sectional surveys of food and beverage intake among national samples of infants, toddlers, and children aged < 4 years living in the 50 U.S. states and Washington, D.C. This study used data from the two most recent surveys. The first was conducted from June 2008 through January 2009 (FITS 2008) [32], a period that preceded implementation of the 2009 revisions in all of these jurisdictions, with the exception of New York, which implemented the revisions on 3 January 2009 [31]. The second was conducted from June 2015 through May 2016 (FITS 2016) [31], after the implementation of the revisions. Details on the sampling methodology for the two surveys are available elsewhere [31,32].

In both FITS 2008 and FITS 2016, data were collected from the primary caregiver of the child (respondent) using a series of questionnaires administered via mail, telephone, and the internet (the latter in 2016 only) [31,32]. In this study, we used data from two of the questionnaires. The first was the recruitment questionnaire, which collected sociodemographic and lifestyle data about the sampled child and their household. The second was an age-specific feeding practices questionnaire, which collected data about the sampled child’s breastfeeding practices.

### 2.3. Study Participants

The sample for this study was restricted to infants (ages 0–11.9 months) whose birth mothers were FITS respondents, whose household incomes were ≤250% of the FPL, and for whom data on at least one of the three breastfeeding outcomes were available.

### 2.4. Outcome Variables

The outcomes chosen for this study were whether the infant was ever breastfed, breastfed through 6 months, and exclusively breastfed through 6 months, consistent with the recommendations of the American Academy of Pediatrics [35] and Healthy People 2030 [36]. In each instance, the outcome variable was derived from a maternal answer to a question on the infant feeding practices questionnaire. Ever breastfed was reported as a binary variable (yes/no) based on the mother’s response to the question “*Was (insert name of child) ever breastfed or fed breast milk?*” Any breastfeeding through 6 months and exclusive breastfeeding through 6 months were reported as binary variables (yes/no) based on a mother’s answers to the following questions, which were asked only if (1) she responded “yes” to the preceding question and (2) her infant was at least six months old at the time of the infant feeding practices questionnaire: “*Are you currently breast feeding (insert name of child)?*” and “*Are you giving your child anything at all in addition to breast milk? This might include water and/or formula.*”

### 2.5. Independent Variables

The primary independent variables captured the effect of the 2009 WIC food package revisions on breastfeeding outcomes and were defined as follows: We first created a dichotomous treatment variable in which infants eligible for WIC based on household income (≤185% FPL) were coded as 1 (treatment group) and infants whose household incomes were just above the WIC eligibility threshold (>185% FPL–250% FPL) were coded as 0 (control group). We measured FPL by examining the FITS’ categorical income variable, which we first converted to a continuous form by assigning the midpoint of the interval of each category [37], and FITS’ household size variable. We next created a dichotomous post-treatment variable in which infants who participated in FITS 2016 were coded as 1 and infants who participated in FITS 2008 were coded as 0. The interaction between the treatment variable and the post-treatment variable captured the difference in the effect of the 2009 revisions on WIC-eligible infants relative to infants who were similar in observable and unobservable characteristics except for their inability to participate in WIC.

The other independent variables we included in our analysis were the infant’s sex and race/ethnicity; whether the infant was born with a low birth weight; whether the infant attended daycare; the mother’s age, race/ethnicity, education, and marital status; whether the infant was the mother’s first; whether the mother had worked in the past 30 days; and the size of the infant’s household.

### 2.6. Statistical Analysis

We calculated weighted and unweighted descriptive statistics to demonstrate the characteristics of the FITS sample. For each outcome, we used a multivariable linear regression model (also referred to as a “linear probability model”) with robust standard errors to facilitate the interpretation of the results [38]. Each model took the following form:

$$Y_{it}=\beta_{0}+\beta_{1}\left(treat_{i}\right)\;+\;\beta_{2}\left(post_{t}\right)\;+\;\beta_{3}\left(treat_{i}\times post_{t}\right)+\beta_{4}X_{it}+\varepsilon_{it}$$

where *Y_it_* is an outcome for infant *i* in year *t*; *treat_i_* is a dichotomous indicator equal to 1 if the infant was in the treatment group and 0 if the infant was in the control group; *post_t_* is a dichotomous indicator equal to 0 if the infant was in the FITS 2008 cohort and 1 if the infant was in the FITS 2016 cohort; and *X_it_* is a vector of covariates for which balance was not achieved (the metric used to assess balance is described below) and were therefore included in the model to account for potential residual confounding [39]. The coefficient for the interaction term, *β*_3_, is the DID estimate of the effect of the 2009 WIC food package revisions on a breastfeeding outcome. It represents the change in outcome before and after the implementation of the 2009 WIC food package revisions in the treatment group (reflecting the true effect of the revision *plus* secular trends in the outcome) minus the change in outcome before and after the implementation of the 2009 WIC food package revisions in the control group (reflecting secular trends in the outcome).

We estimated propensity score weights in accordance with the technique described by Stuart et al. [33]. First, we classified the sample infants into four groups: group 1 = treatment, 2008 FITS; group 2 = control, 2008 FITS; group 3 = treatment, 2016 FITS; and group 4 = control, 2016 FITS. We then used a logistic regression model to estimate a propensity score for each participant, defined as the probability of being in group 1 given a set of observed baseline covariates associated with exposure to WIC’s food packages and/or breastfeeding outcomes [30]. Covariates that met this criterion and thus were included in the propensity score model were the independent variables other than the primary independent variables described in Section 2.5. Following the estimation of the propensity scores, we calculated an inverse probability of treatment weight for each observation reflecting the covariate distribution in group 1 [33]. For each pair of groups (i.e., group 1 and group 2, group 1 and group 3, and group 1 and group 4), we dropped observations with propensity scores that fell outside the range of overlapping propensity scores across treatment and control groups (“common support”). Then, we assessed the balance of the covariate distribution using an inspection of graphs and a calculation of absolute standard mean differences (ASMDs). We considered a weighted ASMD < 0.10 to indicate an acceptable degree of balance [33].

Statistical analyses were performed using SAS Viya 3.04, release 5.2.3. Alpha was set to 0.05. This study was approved as an exempt study by the George Mason University Institutional Review Board due to its use of de-identified data.

## 3. Results

### 3.1. Descriptive Statistics

Weighted descriptive statistics are shown in Table 1, and unweighted descriptive statistics are shown in Supplemental Table S1. A total of 1,114 infants were included in the analysis, of whom 325 (29.2%) participated in FITS 2008 and 789 (70.8%) participated in FITS 2016. Prior to weighting, there were significant differences between the treatment group and the control group in each of the survey cohorts: In both FITS 2008 and FITS 2016, mothers and infants in the control group were more likely to be white and live in smaller households, and mothers in the control group were more likely to have completed more years of schooling and be married or living with a partner compared to mothers in the treatment group (Supplemental Table S1). In addition, among participants in FITS 2016 (but not FITS 2008), mothers in the control group were generally older than mothers in the treatment group. These differences suggest that infants in the control group in both FITS 2008 and FITS 2016 were more socioeconomically advantaged than those in the treatment group, highlighting the importance of incorporating propensity score weighting in the analysis to make these groups more similar. Indeed, after weighting, these differences between the treatment group and the control group in both FITS 2008 and FITS 2016 were no longer present (Table 1).

### 3.2. Balance Assessment

Table 2 presents the results of the ASMDs before and after weighting with the propensity scores. On average, well over half of the unweighted covariates across the three comparison groups showed evidence of imbalance before weighting based on an ASMD of less than 0.10 [33]. After weighting, all of the covariates in comparison 2 showed balance. All but two of the covariates in comparison 1 and three of the covariates in comparison 3 showed balance based on a 0.10 threshold. Covariates for which balance was not achieved after weighting in any of the comparison groups were included in the DID linear probability models to account for potential residual confounding [39].

### 3.3. DID Results

Table 3 shows the adjusted changes in outcomes among the two infant groups for each of the breastfeeding outcomes. The probability of an infant in the treatment group ever breastfeeding was 74% in 2008 and 84% in 2016, reflecting a statistically significant increase of 10 percentage points (*p* < 0.01). Likewise, the probability of an infant in the control group ever breastfeeding was 78% in 2008 and 93% in 2016, reflecting a statistically significant increase of 15 percentage points (*p* < 0.05). However, the adjusted DID estimate for this outcome was not statistically significant (β = 0.05; 95% CI: −0.10, 0.19), indicating no association of the 2009 WIC food package revisions with ever breastfeeding.

There were no statistically significant pre–post differences in breastfeeding through 6 months among infants in either the treatment group or the control group, and the adjusted DID estimate for that outcome was not statistically significant (β = −0.17; 95% CI: −0.46, 0.12), reflecting no association between the 2009 WIC food package revisions and breastfeeding through 6 months.

Similarly, there were no statistically significant pre–post differences in exclusive breastfeeding through 6 months among infants in either the treatment group or the control group, and the adjusted DID estimate for that outcome was not statistically significant (β = 0.03; 95% CI: −0.16, 0.22), reflecting no association between the 2009 WIC food package revisions and exclusive breastfeeding through 6 months.

## 4. Discussion

This study evaluated whether the 2009 WIC food package revisions led to changes in the rates of ever breastfeeding, breastfeeding through 6 months, and exclusively breastfeeding through 6 months by comparing changes in these outcomes before and after the 2009 WIC food package revisions among WIC-eligible infants relative to pre–post changes in the same outcomes among a carefully constructed control group. We found a statistically significant pre–post increase in the rate of ever breastfeeding among the WIC-eligible infants, comparable to the findings in other studies [27,28]. However, this change was not statistically different from a similar change in the same outcome among our control group. There were no pre–post differences in breastfed through 6 months or exclusively breastfed through 6 months in either the treatment group or the control group, nor were the DID estimates for these outcomes statistically significant. Overall, we did not find any evidence of increases in any of our three breastfeeding outcomes among infants in the treatment group relative to infants in the control group. Thus, any increases in rates of ever breastfeeding and breastfeeding through 6 months among the WIC-income-eligible infants appear to be reflections of secular trends in breastfeeding and not the result of the 2009 WIC food package changes.

The reason for the 2009 revisions’ apparent lack of effect on breastfeeding behaviors is unclear. One possibility may be the perceived inadequacy of the economic incentive to breastfeed that the revisions sought to create. There are two ways to conceptualize this incentive. The first is to consider the incentives from the food packages for the mother and the infant in tandem since both are simultaneously eligible to receive them. This was the approach utilized by the IOM in 2005 [19] and the USDA in 2009 [20]. According to the IOM’s data, the pre-2009 annualized market values of the food packages for the fully formula-feeding and partially formula-feeding mother–infant dyads were, respectively, 107% and 150% greater than the market value of the food package for the fully breastfeeding mother–infant dyads (Supplemental Table S2). After the 2009 revisions, these differentials were estimated to be 31% and 10%, respectively, reflecting a significant narrowing of the gaps in market values (Supplemental Table S2). Nevertheless, based on the IOM’s own reasoning, the failure to fully eliminate these gaps could mean that women still perceive the food packages with formula to be the more attractive option.

An alternative way to conceptualize the incentive is to focus solely on the value of the food packages for breastfeeding mothers. This is also a reasonable approach since the formula acts as a substitute for the mother’s own breast milk and has no other value to her beyond this substitution. For the mother to forego receiving formula (whether in whole or in part) by choosing to breastfeed, an incentive would need to be offered that provides a benefit to her that exceeds the costs of her breastfeeding. In theory, the maternal food packages provide that incentive. After the 2009 revisions, the annualized market value of the food package for fully breastfeeding women increased by nearly 13% (Supplemental Table S2). However, breastfeeding rates did not improve as a result of the revisions. A standard economic assumption is that, in the aggregate, a population responds to changes in economic incentives. Since that did not happen here, perhaps this incentive was not sufficient to elicit the desired response. This suggests, in turn, that there is a perceived cost to breastfeeding, and the additional incentive reflected in the increased value of the post-2009 fully breastfeeding food package was insufficient to offset that cost. In this context, costs may take the form of barriers to breastfeeding. Research has documented numerous barriers to breastfeeding experienced by WIC participants, including a lack of familial support [40,41]; discomfort with breastfeeding in public [41]; unsupportive supervisors and coworkers [41,42], a lack of private pumping space while at work [41,42], and physical issues such as pain and discomfort while breastfeeding [41,43].

It should be noted that in 2017, in response to a congressional mandate [44], the National Academy of Sciences, Engineering, and Medicine (NASEM; IOM’s successor organization) issued an updated set of recommendations for revising the WIC food packages that was consistent with the current nutritional guidelines, the health and cultural needs of the WIC participant population, and the goal of cost neutrality [25]. Interestingly, in this round of recommendations, NASEM did not continue with its earlier stance of focusing on the market values of the mother–infant dyad food packages to encourage breastfeeding. Instead, it explained that the benefit of the breastfeeding food packages encompasses more than the foods provided by them and includes other WIC services, in particular WIC’s peer-counseling program [25]. Studies have shown that this program, which connects WIC participants with other current or former WIC participants in their community who have personal breastfeeding experience and are trained to provide breastfeeding information and support, is effective in improving both the initiation rate and duration of breastfeeding among the WIC population [45,46,47,48]. NASEM urges the communication of this benefit to WIC participants as a means of increasing the perceived value of the breastfeeding food packages [25].

A key strength of this study is its design, which combined a DID model with an ITT analysis and propensity score weighting. An ITT analysis is appropriate for evaluating government programs that offer benefits that are conditional upon enrollment, such as WIC. Since not every person eligible for the program decides to enroll, an ITT analysis estimates the difference in outcomes between the people offered the program (i.e., the treatment group) and the people not offered the program (i.e., the control group), regardless of whether the people offered the program in fact enrolled in it. This approach reduces the potential for selection bias that occurs when people who choose to enroll in a program differ systematically from people who choose not to enroll. More importantly, from a policy standpoint, an ITT analysis provides information about the effect of offering a program, which typically is of more interest to policy makers, who can offer a program but cannot compel people to enroll in it. We reduced the potential for selection bias even further with the use of propensity score weighting. The DID model allowed us to distinguish overall trends in outcomes from the effect of the WIC food package policy change on those outcomes.

The results of this study must be interpreted in light of its limitations. One such limitation is its small sample size, which may have resulted in inadequate power to detect pre- and post-2009 differences in our breastfeeding outcomes. However, given that our results were generally consistent with other studies that have examined this issue, one of which used two datasets consisting of approximately 127,000 and 74,000 observations [27], this limitation may be less critical than it would otherwise be in the absence of any prior research. Another limitation is the lack of precision with respect to several key variables. For example, both FITS 2008 and FITS 2016 collected income data as a categorical variable, which we converted to a continuous variable. This created the potential for measurement error in the identification of the treatment and control groups based on FPL calculations. Additionally, breastfeeding outcomes as well as income and other covariates were self-reported and may suffer from reporting bias. In the case of breastfeeding outcomes, however, research has shown that maternal recall of infant feeding practices tends to be highly reliable, especially if the recall occurs within a few years after childbirth [49,50]. The treatment group was defined solely in terms of the eligibility for WIC based on household income and did not take into account the requirement that applicants also be at “nutritional risk” [51]. However, nearly all persons who are income-eligible for WIC also satisfy at least one of the nutritionally at-risk criteria [52]. Finally, New York implemented the food package revisions on January 3, 2009, whereas FITS 2008 data were collected until the end of January 2009 [53]. This raises the potential for a New York-based FITS 2008 infant whose data were collected after 3 January 2009 to have been exposed to the 2009 revisions, although we believe this potential to be quite small.

Several limitations relate to the assumptions of the DID model. The first of these is the parallel trends assumption, which requires that outcome differences between the treatment and control groups be constant over time [34]. Because our data were limited to two time periods, we could not test this assumption directly. However, we assessed the plausibility of this assumption by visually examining pretreatment trends in breastfeeding behaviors reflected in other data. Using estimates that we obtained from the CDC Division of Nutrition, Physical Activity, and Obesity’s “Nutrition, Physical Activity, and Obesity: Data, Trends, and Maps” interactive database [54], we constructed plot graphs showing annual rates of each of our breastfeeding outcomes from 2004 to 2016, stratified by three income groups that overlapped with the income groups defining our study’s treatment and control groups (Supplemental Figure S1). As can be observed in Supplemental Figure S1, each of our breastfeeding outcomes had similar upward trajectories among the three income groups prior to 2009 and in fact became parallel in 2008. These estimates thus provide indirect support for the plausibility of the parallel trends assumption for our analysis.

The second assumption we needed to meet in order to apply our DID model was the absence of any other event occurring between 2009 and 2016 that affected the breastfeeding outcomes of the treatment group differently from those of the control group [34]. One possibility was the 2010 enactment of the Patient Protection and Affordable Care Act, which included provisions intended to make breastfeeding more accessible and affordable for American women [55]. However, there is no indication that these provisions impacted WIC-eligible infants and their mothers differently from marginally WIC-ineligible infants and their mothers, given that in most states during this period Medicaid covered various breastfeeding support services during pregnancy and for the duration of breastfeeding [56]. In sum, it is reasonable to conclude that there were no post-2009 events that differentially impacted breastfeeding rates among our comparison groups.

Finally, the use of propensity scores to address selection bias is contingent on the assumption that all confounders of treatment selection and the outcome have been measured correctly and included in the model. Although it is impossible to conclude definitively that this assumption has been met, its plausibility is greater when a large number of confounders have been included in a model. In this study, we used a rich set of covariates that are associated with both WIC participation and breastfeeding outcomes. Combined with our use of an ITT analysis, the potential for residual bias in our estimates was reduced.

## 5. Conclusions

Our study did not find evidence that the 2009 WIC food package revisions had an effect on ever breastfeeding, breastfeeding through 6 months, or exclusively breastfeeding through 6 months among a sample of infants eligible for WIC based on household income. Any positive effects observed in this study and prior studies that assessed this relationship may be reflections of the upward trends in breastfeeding rates that occurred in the U.S. before and after the implementation of the revisions.

## Figures and Tables

**Table 1:** Weighted descriptive characteristics of 1114 infants participating in FITS 2008 and FITS 2016.

Measures		FITS 2008			FITS 2016		
		Treatment ^2^	Control ^3^	*p* ^4^	Treatment ^2^	Control ^3^	*p* ^4^
Outcomes (weighted ^1^ %)							
Ever breastfed (y) (*n* = 1114)		40.7	33.7	0.52	46.8	40.5	<0.01
Breastfed through 6 months (y) (*n* = 521)		19.8	26.7	0.31	26.9	20.9	0.32
Breastfed exclusively through 6 months (y) (*n* = 257)		4.3	3.6	0.75	3.7	2.7	0.93
Characteristics (weighted ^1^ % or m (sd))		(*n* = 227)	(*n* = 98)		(*n* = 634)	(*n* = 155)	
Infant’s sex				0.88			0.96
	Male	32.0	24.2		30.8	23.6	
	Female	24.3	19.4		26.0	19.6	
Infant’s race/ethnicity				0.86			0.14
	White ^5^	30.6	25.8		31.1	26.9	
	African American ^5^	8.9	4.2		8.8	2.5	
	Hispanic or Latino	11.9	9.1		11.7	11.7	
	Other ^6^	5.0	4.6		5.1	2.0	
Infant was low birth weight ^7^ (y)		4.5	3.8	0.87	4.5	4.5	0.63
Mother’s age				0.69			0.30
	less than 20 years	5.2	2.3		6.1	1.1	
	20–24 years	15.9	13.9		16.4	10.4	
	25–29 years	15.7	10.2		14.9	12.4	
	30–34 years	11.2	11.1		11.2	12.7	
	35–39 years	6.2	5.5		6.0	5.4	
	40 years or more	2.2	0.6		2.3	1.2	
Mother’s race/ethnicity				0.68			0.08
	White ^5^	37.5	32.7		38.4	34.5	
	African American ^5^	8.7	4.2		8.5	2.6	
	Hispanic or Latino	8.7	6.3		8.4	5.5	
	Other ^6^	1.5	0.4		1.5	0.6	
Mother’s education				0.57			0.30
	Attended high school/received high school diploma	29.6	18.8		29.5	17.7	
	Attended college/received college degree	24.6	22.2		24.9	23.6	
	Attended graduate school/received graduate degree	2.2	2.6		2.4	1.9	
Mother’s marital status				0.25			0.28
	Married/living with partner	40.2	37.3		41.9	36.5	
	Never married	3.2	1.4		3.3	1.1	
	Separated/divorced/widowed	12.9	5.0		11.7	5.6	
Infant is mother’s first child (y)		18.6	14.7	0.94	18.9	11.8	0.48
Household size		4.8 (1.6)	4.6 (1.6)	0.16	4.8 (1.1)	4.8 (1.8)	0.94
Mother worked in last 30 days (y)		20.1	18.3	0.46	19.6	17.9	0.36
Infant attends daycare (y)		14.2	11.4	0.88	13.7	8.6	0.54

Abbreviations: FITS, Feeding Infants and Toddlers Study; FPL, federal poverty level; m, mean; *n*, sample size; sd, standard deviation; WIC, Special Supplemental Nutrition Program for Women, Infants, and Children; y, yes. ^1^ All statistics are weighted using propensity scores, with the exception of the n’s, which are unweighted to show sample sizes. ^2^ The treatment group consisted of sample infants who were eligible for WIC based on household income (≤185% FPL). ^3^ The control group consisted of sample infants who were ineligible for WIC based on a household income just above the WIC eligibility threshold (>185% FPL—250% FPL). ^4^ Chi-square for categorical variables; independent-samples t-test for continuous variable. ^5^ Data do not distinguish between Hispanic/Latino and non-Hispanic/Latino ethnicity. ^6^ American Indian and Alaska Native, Asian, Native Hawaiian, and Other Pacific Islander infants are categorized as other. ^7^ Low birth weight is < 2500g.

**Table 2:** Absolute standardized mean differences of sample characteristics before and after weighting.

		Group 1 (2008 Treatment vs. 2008 Control)		Group 2 (2008 Treatment vs. 2016 Treatment)		Group 3 (2008 Treatment vs. 2016 Control)	
Covariate		ASMD before Weighting	ASMD after Weighting	ASMD before Weighting	ASMD after Weighting	ASMD before Weighting	ASMD after Weighting
Propensity score		1.375 ^1^	0.013	0.652 ^1^	0.009	1.671 ^1^	0.075
Infant is female		0.010	0.013	0.134 ^1^	0.051	0.028	0.049
Infant’s race/ethnicity ^3^							
	White	0.722 ^1^	0.027	0.164 ^1^	0.013	0.349 ^1^	0.003
	Black	0.503 ^1^	0.054	0.004	0.009	0.264 ^1^	0.030
	Hispanic/Latino	0.320 ^1^	0.089	0.065	0.013	0.148 ^1^	0.108 ^2^
	Other	0.252 ^1^	0.123 ^2^	0.224 ^1^	0.009	0.095	0.126 ^2^
Infant was low birth weight ^3^		0.083	0.089	0.003	0.002	0.064	0.113 ^2^
Mother’s race/ethnicity							
	White	0.570 ^1^	0.019	0.040	0.021	0.224^1^	0.080
	Black	0.492 ^1^	0.054	0.000	0.011	0.232^1^	0.047
	Hispanic/Latino	0.276 ^1^	0.042	0.008	0.017	0.192^1^	0.045
	Other	0.047	0.063	0.088	0.001	0.177^1^	0.028
Mother’s age ^3^							
	less than 20 years	0.172 ^1^	0.050	0.334 ^1^	0.064	0.409 ^1^	0.026
	20–24 years	0.231 ^1^	0.079	0.124 ^1^	0.015	0.522 ^1^	0.031
	25–29 years	0.030	0.063	0.033	0.034	0.225 ^1^	0.048
	30–34 years	0.174 ^1^	0.047	0.139 ^1^	0.003	0.305 ^1^	0.075
	35–39 years	0.210 ^1^	0.027	0.143 ^1^	0.013	0.098	0.001
	40 years or more	0.119 ^1^	0.125 ^2^	0.037	0.001	0.056	0.077
Mother’s education							
	Attended high school/received diploma	0.464 ^1^	0.076	0.417 ^1^	0.011	0.882 ^1^	0.001
	Attended college/received degree	0.460 ^1^	0.036	0.431 ^1^	0.006	0.790 ^1^	0.025
	Attended graduate school/received graduate degree	0.004	0.098	0.044	0.015	0.105 ^1^	0.053
Mother’s marital status							
	Married/living with partner	0.595 ^1^	0.019	0.203 ^1^	0.054	0.682 ^1^	0.067
	Never married	0.539 ^1^	0.025	0.170 ^1^	0.059	0.600 ^1^	0.074
	Separated/divorced/widowed	0.198 ^1^	0.080	0.092	0.001	0.253 ^1^	0.003
Infant is mother’s first child		0.021	0.082	0.117 ^1^	0.004	0.010	0.077
Household size		0.349 ^1^	0.079	0.044	0.022	0.437 ^1^	0.084
Mother worked in last 30 days		0.124 ^1^	0.081	0.046	0.026	0.042	0.086
Child attends daycare		0.215 ^1^	0.011	0.081	0.024	0.053	0.058

Abbreviations: ASMD, absolute standardized mean difference; Control, infants in the control group (household income >185–250% federal poverty level); DID, difference-in-differences; Treat, infants in the treatment group (household income ≤185% federal poverty level). ^1^ASMD ≥0.10 before weighting by propensity scores. ^2^ ASMD ≥ 0.10 after weighting by propensity scores. ^3^Covariate included in DID linear probability models to account for potential residual confounding.

**Table 3:** Difference-in-differences estimates ^1^.

Comparison Groups		Ever Breastfed (*n* = 1114)	Breastfed through 6 Months (*n* = 521)	Breastfed Exclusively through 6 Months (*n* = 257)
Treatment Group (WIC-eligible)				
	2008, mean (rSE)	0.74 (0.04)	0.20 (0.07)	0.08 (0.07)
	2016, mean (rSE)	0.84 (0.04)	0.27 (0.06)	0.05 (0.05)
	First difference, row 3–row 2 (95% CI)	0.10 (0.03, 0.17) **	0.07 (−0.05, 0.19)	−0.03 (−0.14, 0.07)
Control Group (marginally WIC-ineligible)				
	2008, mean (rSE)	0.78 (0.07)	0.35 (0.09)	0.06 (0.07)
	2016, mean (rSE)	0.93 (0.04)	0.26 (0.12)	0.06 (0.05)
	Second difference, row 7–row 6 (95% CI)	0.15 (0.02, 0.28) *	−0.10 (−0.36, 0.17)	−0.001 (−0.17, 0.17)
WIC policy change effect ^2^, row 8–row 4 (95% CI)		0.05 (−0.10, 0.19)	−0.17 (−0.46, 0.12)	0.03 (−0.16, 0.22)

Abbreviations: CI, confidence interval; DID, difference-in-differences; FITS, Feeding Infants and Toddlers Survey; FPL, federal poverty level; rSE, robust standard error; WIC, Special Supplemental Nutrition Program for Women, Infants, and Children. ^1^ Estimates were obtained using linear probability models for binary outcomes with robust standard errors and propensity score weights. Infants’ race/ethnicity data, whether infants had low birth weights (<2500 g), and mothers’ ages were included in the models to control for residual confounding. Sample sizes for breastfed through 6 months and breastfed exclusively through 6 months were smaller than that of ever breastfed due to missing values. Coefficients and 95% confidence intervals are reported for differences in each breastfeeding outcome between 2008 and 2016 among the treatment group and the control group and for the DID estimate. For all other estimates, the coefficients and robust standard errors are reported. Any difference errors are due to rounding. ^2^ The DID estimate, which represents the coefficient for the interaction between the treatment variable (household income < 185% of FPL, coded as 1, and household income of 185% FPL–250% FPL, coded as 0) and the post-treatment variable (data from FITS 2016, coded as 1, and data from FITS 2008, coded as 0). * *p* < 0.05, ** *p* < 0.01.

## Data Availability

The Feeding Infants and Toddlers Study data are proprietary. Access was granted by Nestlé Research (Societé des Produits Nestlé, Lausanne, Switzerland) to the authors for the purpose of this research.

## Supplementary Materials

The following supporting information can be downloaded at: https://www.mdpi.com/article/10.3390/nu15020414/s1, Table S1: Unweighted descriptive characteristics of 1114 infants participating in FITS 2008 and FITS 2016; Table S2: Estimated annualized market values of WIC food packages before and after the 2009 WIC food package revisions; Figure S1: National breastfeeding rates, stratified by federal poverty level (FPL) categories.
