# Supplementary material for: A Quasi-Experimental Assessment of the Effect of the 2009 WIC Food Package Revisions on Breastfeeding Outcomes
Source: Nutrients. 2023 Jan 13;15(2):414. doi: 10.3390/nu15020414 (PMC9862204; doi:10.3390/nu15020414)

Supplemental Table S1. Unweighted descriptive characteristics of 1,114 infants participating in FITS 2008 and FITS 2016

Measures	FITS 2008			FITS 2016		
	Treatment[1]	Control[2]	p[3]	Treatment[1]	Control[2]	p[3]
**Outcomes (%)**						
Ever breastfed (y) (n=1,114)	72.3	78.6	0.23	81.7	92.3	<0.01
Breastfed through 6 months (y) (n=521)	40.5	53.9	0.13	50.8	61.3	0.10
Breastfed exclusively through 6 months (y) (n=257)	9.4	4.4	0.63	7.1	8.3	0.76
**Characteristics (% or m (sd))**	(n=227)	(n=98)		(n=634)	(n=155)	
Infant's sex			0.91			0.47
Male	56.8	56.1		50.3	53.6	
Female	43.2	43.9		49.7	46.5	
Infant's race/ethnicity			<0.001			<0.001
White[4]	60.1	74.3		60.1	74.3	
African-American[4]	15.8	5.5		15.8	5.5	
Hispanic or Latino	19.3	14.6		19.3	14.6	
Other[5]	4.9	5.5		4.9	5.5	
Infant was born with low birth weight[6] (y)	7.9	5.1	0.36	8.0	5.8	0.35
Mother's age			0.12			<0.01
<20 years	9.3	5.1		1.7	0.7	
20 – 24 years	28.2	19.4		22.9	9.7	
25 - 29 years	27.8	27.6		29.2	36.1	
30 – 34 years	19.8	28.6		25.7	35.5	
35 – 39 years	11.0	17.4		15.8	13.6	
40 years or more	4.0	2.0		4.7	4.5	
Mother's race/ethnicity			<0.001			0.01
White[4]	66.5	88.8		64.8	74.2	
African-American[4]	15.4	2.0		15.5	8.4	
Hispanic or Latino	15.4	7.1		15.8	10.3	
Other[5]	2.6	2.0		3.9	7.1	
Mother's education			<0.01			<0.001
Attended high school/received high school diploma	52.4	31.6		32.3	16.1	
Attended college/received college degree	43.6	64.3		64.5	76.8	
Attended graduate school/received graduate degree	4.0	4.1		3.2	7.1	
Mother's marital status			<0.001			<0.001
Married/living with partner	71.4	92.9		80.0	94.8	
Never married	5.7	2.0		3.8	1.3	
Separated/divorced/widowed	22.9	5.1		16.3	3.9	
Infant is mother's first child (y)	33.0	31.6	0.80	27.6	27.1	0.90
Household size	4.8 (1.6)	4.3 (1.0)	<0.01	4.7 (1.7)	4.3 (1.2)	<0.001
Mother worked in last 30 days (y)	35.7	39.8	0.48	33.4	36.8	0.43
Infant attends daycare (y)	25.1	31.6	0.22	21.6	21.3	0.93

Abbreviations: FITS, Feeding Infants and Toddlers Study; FPL, federal poverty level; m, mean; n, sample size; sd, standard deviation; WIC, Special Supplemental Nutrition Program for Women, Infants, and Children; y, yes.

[1]The treatment group consists of sample infants who were eligible for WIC based on household income (≤185% FPL). [2]The control group consists of sample infants who were ineligible for WIC based on household income just above the WIC eligibility threshold (>185% FPL – 250% FPL). [3]Chi-square or Fisher's exact test for categorical variables; independent samples t-test for continuous variable. [4]Data do not distinguish between Hispanic/Latino and non-Hispanic/Latino ethnicity. [5]American Indian and Alaska Native, Asian, Native Hawaiian or Other Pacific Islander infants are categorized as Other. [6]Low birth weight is < 2,500 grams.

Supplemental Table S2. Estimated annualized market values of WIC food packages before and after the 2009 WIC food package revisions

	Pre $[1]	Post $	Pre/Post $ Δ	Pre/Post % Δ	Pre-Revisions Gap		Post-Revisions Gap	
					$	%	$	%
**Maternal/Infant Dyad Food Packages**								
Fully breastfeeding	$668	$1,028	$360	53.9%	ref	ref	ref	ref
Partially breastfeeding	$1,669	$1,135	-$534	-32.0%	$1,001	149.9%	$107	10.4%
Fully formula feeding	$1,380	$1,344	-$36	-2.6%	$712	106.6%	$316	30.7%
**Maternal Food Packages**								
Fully breastfeeding	$607	$685	$78	12.9%	ref	ref	ref	ref
Partially breastfeeding	$495	$110	-$385	-77.8%	-$112	-18.5%	-$575	-83.9%
Fully formula feeding	$206	$224	$18	8.7%	-$401	-66.1%	$461	67.3%

[1]Market value amounts (in dollars) were obtained from Table 5.5 (pp. 142-143) in Chapter 5 of the IOM report *WIC Food Packages: Time for a Change*[19].

Figure S1. National breastfeeding rates, stratified by federal poverty level (FPL) catergories

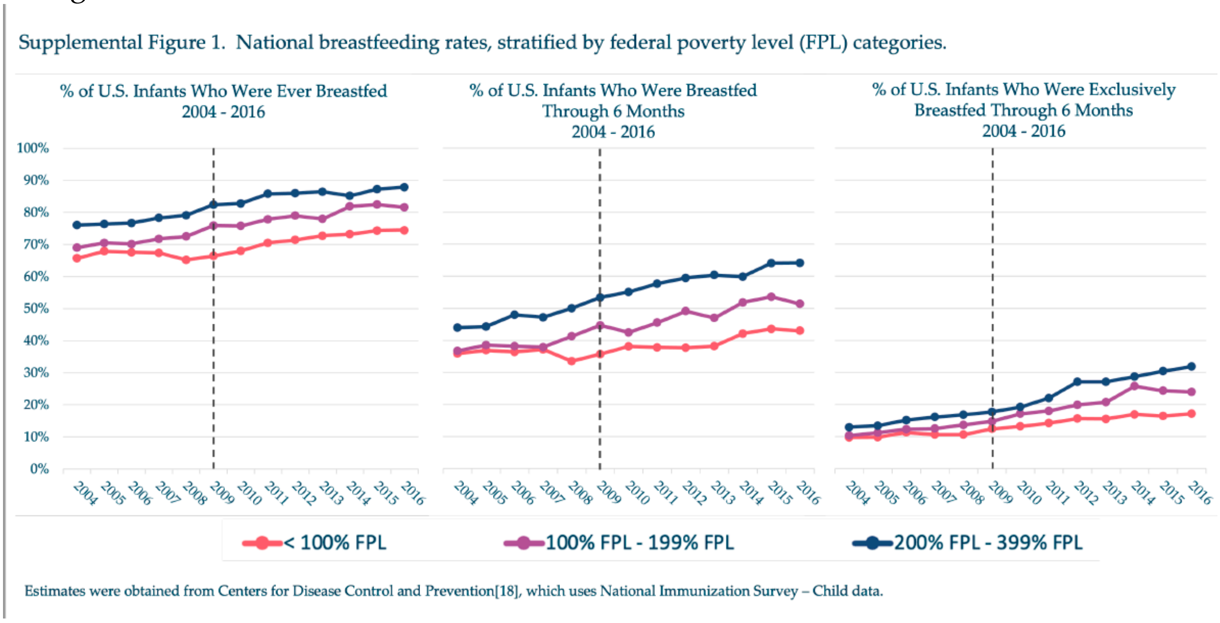

Supplement: Supplementary file 1 [file nutrients-15-00414-s001.zip › nutrients-2131115-supplementary material.pdf]
